# Potential interactive effects between invasive *Lumbricus terrestris* earthworms and the invasive plant *Alliaria petiolata* on a native plant *Podophyllum peltatum* in northeastern Ohio, USA

**DOI:** 10.1093/aobpla/plaa073

**Published:** 2020-12-29

**Authors:** Colin G Cope, Sarah R Eysenbach, Alexandra S Faidiga, Constance E Hausman, Juliana S Medeiros, Jennifer E Murphy, Jean H Burns

**Affiliations:** 1 Department of Biology, Case Western Reserve University, Cleveland, OH, USA; 2 The Holden Arboretum, Kirtland, OH, USA; 3 Cleveland Metroparks, Parma, OH, USA

**Keywords:** context-dependence, interactive effects, invasion, invasive earthworms, invader impacts, physiology

## Abstract

We test whether the invasive earthworm *Lumbricus terrestris* and leaf litter of the invasive herbaceous plant *Alliaria petiolata* interact to influence the native plant, *Podophyllum peltatum*, using both observational field data and a multi-year experiment. We hypothesized invader interactive effects on the native plant might result from either changes in allelochemical distribution in the soil or nutrient availability mediated by the invasive earthworm pulling leaf litter down into the soil. Within the field data we found that *Alliaria petiolata* presence and higher soil nitrogen correlated with reduced *Podophyllum peltatum* cover, and no evidence for an invader–invader interaction. Within the factorial experiment, we found a super-additive effect of the two invaders on plant biomass only when activated carbon was present. In the absence of activated carbon, there were no differences in *Podophyllum peltatum* biomass across treatments. In the presence of activated carbon, *Podophyllum peltatum* biomass was significantly reduced by the presence of both *Lumbricus terrestris* and *Alliaria petiolata* leaf litter. The absence of an effect of *Alliaria petiolata* leaves without activated carbon, combined with a failure to detect arbuscular mycorrhizal colonization, suggests that indirect effects of allelochemicals on arbuscular mycorrhizal fungi were not the primary driver of treatment responses. Rather direct nutrient availability might influence a potential interaction between these invaders. Leaf nitrogen content was higher and leaf CO_2_ concentration was lower in the presence of *Lumbricus terrestris*, but treatment did not influence maximum photosynthetic rate. While the field data do not suggest a negative interaction between these invaders, the experiment suggests that such an interaction is possible with greater environmental stress, such as increasing nitrogen deposition. Further, even plants with rapid physiological responses to increased nitrogen availability may have other physiological limits on growth that prevent them from compensating from the harm caused by multiple invaders.

## Teaser

Invasive earthworms (*Lumbricus terrestris*) may magnify the negative effects of garlic mustard leaves (*Alliaria petiolata*), but only under some soil conditions.

## Introduction

**Figure 1.**
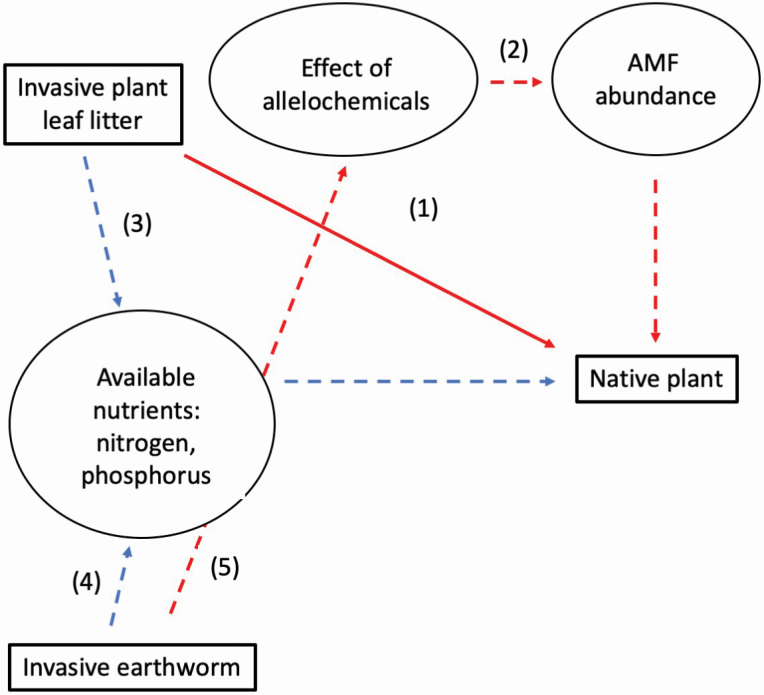
The leaf litter of invasive plants, such as *Alliaria petiolata*, may negatively influence native plant growth via allelochemicals, (1) which may have direct phytotoxic effects on the native plant, or (2) which may suppress plant mutualists, such as arbuscular mycorrhizal fungi (AMF), indirectly suppressing native plants. (3) *Alliaria petiolata* leaf litter can also influence nutrient availability in the soil. Invasive earthworms can also influence native plants by enhancing nutrient (e.g. nitrogen, phosphorus) availability in the soil, which could (4) interact with invader leaf nutrient input (e.g. via changes in litter decomposition), or could (5) shift the distribution or rate of allelochemical input in the soil. Putative direct effects on native plants are shown with solid arrows and indirect with dashed arrows. Putative negative effects are shown in red and positive effects are shown in blue. Circles indicate putative mechanisms that were not directly measured (i.e. ‘latent variables’ in the language of path analysis). See text in the Introduction for additional details.

Assessment of effects of invaders have generally focussed on single invasive species, despite the frequent observation of systems with multiple invaders (Kuebbing and Nunez 2015). Interactions between multiple invaders could be additive (e.g. Milton *et al*. 2007; Cushman and Gaffney 2010; Shaben and Myers 2010; Braga *et al*. 2019), sub-additive (Yang *et al*. 2011) or super-additive (Green *et al*. 2011) and mostly focus on plant–plant interactions (Kuebbing and Nunez 2015). We propose that invasive earthworms, like *Lumbricus terrestris* (Lumbricidae), might interact with invasive plants because of their feeding mode of pulling leaf litter down into the soil (Edwards 2004; Fahey *et al*. 2013), potentially redistributing allelochemicals and nutrients produced by invasive plant leaf litter (Fig. 1). For example, *Alliaria petiolata* leaf litter produces allelochemicals that suppress plant mutualists belowground (Cantor *et al*. 2011). Invader interactions belowground might particularly influence plant physiology, because photosynthetic rates are limited by phosphorus availability (Rychter and Rao 2005), and phosphorus uptake is mediated by arbuscular mycorrhizal mutualists in many cases. Photosynthetic rates are also limited by nitrogen availability, which is a component of RuBisCO, the enzyme that fixes carbon in C_3_ plants (von Caemmerer and Quick 2000). By partnering field and experimental data, we explore this potential interaction between invasive earthworms and *Alliaria petiolata* leaf litter under realistic field conditions, as well as characterizing physiological mechanisms in a manipulative experiment.

The invasive plant, *Alliaria petiolata*, can directly suppress native plants via competition (Barto and Cipollini 2009), but here we focus on potential effects via leaf litter (Fig. 1). The leaf litter of *Alliaria petiolata* is known to have strong negative effects on native plants, through the production of glucosinolate and flavonoid allelochemicals (Hale and Kalisz 2012) and changes in nutrient cycling (Rodgers *et al*. 2008). *Alliaria petiolata* leaf litter and its chemical leachates can have direct phytotoxic effects on native plants (Fig. 1, arrow 1) (Cipollini *et al*. 2012). In addition, *Alliaria petiolata* is known to suppress arbuscular mycorrhizal fungi (AMF) (Fig. 1, arrow 2) (Hale and Kalisz 2012; Cantor *et al*. 2011; Hale *et al*. 2016) and ectomycorrhizal fungi (Wolfe *et al*. 2008), which help plants acquire nutrients from the soil (Mohammadi *et al*. 2011). When *Alliaria petiolata* allelochemicals kill AMF mutualists, this lowers photosynthetic rates in the native plant *Maianthemum racemosum* (Hale *et al*. 2011, 2016). Isolating effects of allelochemicals is methodologically complex and at times controversial (Lau *et al*. 2008; Weisshuhn and Prati 2009). For example, activated carbon is often used in allelopathy studies to absorb allelochemicals (Callaway and Aschehoug 2000; Prati and Bossdorf 2004), but has multiple effects on the soil, increasing the availability of some nutrients (e.g. nitrate) and decreasing others (e.g. ammonium) (Lau *et al*. 2008). While this method cannot by itself determine whether allelopathy occurs, it does change the context of the soil, allowing us to ask whether soil context might influence *Alliaria petiolata* leaf litter effects. *Alliaria petiolata* leaf litter can also alter nutrient cycling (Fig. 1, arrow 3) (Rodgers *et al*. 2008), potentially increasing nutrient availability to native plants.

*Lumbricus terrestris* is an invasive earthworm (Eisenhauer *et al*. 2007), which is likely to interact with *Alliaria petiolata*, because it feeds by pulling leaf litter down into the soil (Zicsi *et al*. 2011). *Lumbricus terrestris*’ feeding behaviour may break down nutrients in leaves faster than decomposition alone, altering nutrient cycling (Fig. 1, arrow 4) (Costello and Lamberti 2009). Both *Lumbricus terrestris* and *Alliaria petiolata* are native to Europe and were introduced to the USA through European settlers in the 1800s (Nuzzo 1993; Eisenhauer *et al*. 2007) and have become widespread problems throughout much of the Eastern USA, where they often co-occur (Nuzzo *et al*. 2009; Zelles 2012). A meta-analysis has indicated that greater invasive earthworm biomass correlates with greater invasive plant cover, consistent with invasive earthworms facilitating invasive plants (Craven *et al*. 2017). Because of this high degree of co-occurrence, we propose that *Lumbricus terrestris* earthworms may amplify the effects of *Alliaria petiolata* allelopathy through their feeding mechanism of pulling litter into underground burrows. *Lumbricus terrestris* might thus increase the distribution of leaf litter over the roots of native plants, and thus increase the negative allelopathic effects of *Alliaria petiolata* (Fig. 1, arrow 5).

Our aims were to determine whether the invasive earthworm *Lumbricus terrestris* and leaf litter of *Alliaria petiolata* might influence native plant abundance, performance and physiology. We focussed on a common herbaceous plant: *Podophyllum peltatum* (Berberidaceae). We selected native species *Podophyllum peltatum* because it is common in the northeastern USA and co-occurs with the two invaders *Lumbricus terrestris* and *Alliaria petiolata*. In addition, *Podophyllum peltatum* associates with AMF (Soudzilovskaia *et al*. 2020), making it a potential candidate for indirect effects (Fig. 1). First, we utilized a large-scale observational dataset to determine if native *Podophyllum peltatum* abundance correlates with invader abundance in the field. Because nutrient availability in the soil influences plant physiology, we explored whether soil nutrients and invaders might interact to influence *Podophyllum peltatum* abundance. Second, we conducted a factorial experiment to determine effects of these two invaders on *Podophyllum peltatum* growth and physiology. We predicted that *Lumbricus terrestris* might increase the exposure of *Podophyllum peltatum* to allelochemicals produced by *Alliaria petiolata*, resulting in greater suppression of native plant biomass in the presence of both invaders (super-additive effect). If the interaction between the invasive plant and earthworm is mediated by allelochemicals in the soil, we predict the interaction will be stronger in the absence of activated carbon, which binds allelochemicals (Fig. 1, arrow 5). An interaction only in the presence of activated carbon would be more consistent with nutrient-mediated effects (Fig. 1, arrow 4). Alternatively, *Lumbricus terrestris* might enhance nutrient availability for *Podophyllum peltatum*, leading to greater plant resistance to allelochemicals produced by *Alliaria petiolata* (sub-additive effect). If this is the case, we predict that *Lumbricus terrestris* might enhance plant access to soil nitrogen, increasing the ability of the native plant to fix carbon (greater Ci and greater Amax in the presence of *Lumbricus terrestris*).

## Methods

### Plant community assessment program—observational data

We utilized a large (400 plots—187 plots met our criteria, see below) plant community dataset (PCAP) collected over a series of 4 years between 2010 and 2013 by Cleveland Metroparks to explore correlates of *Podophyllum peltatum* abundance (percent cover). Cleveland Metroparks encompasses >23 000 acres with 18 reservations that are located within and surrounding the city of Cleveland, OH. The plots were established using the generalized random tessellation stratified design (Stevens and Olsen 2003), providing a spatially balanced random sample. Sampling procedure followed the North Carolina Vegetation Survey method (Peet *et al*. 2004) and plots were sampled from 11 June to 30 August. While *Podophyllum peltatum* and *Alliaria petiolata* cover may be underestimated towards the end of the sampling season, plots were sampled in a geospatially balanced, stratified random sample to avoid systematic bias (Stevens and Olsen 2003). The plots that were sampled covered a 20 m × 50 m area, in which the percent cover of every plant species was recorded, as well as collecting soil samples for nutrient analysis. The presence or absence of earthworms was recorded by visual assessment, where plots with worms, castings and middens were coded as ‘present’. Field crews consisted of 2 teams with typically 4 crew members each. Each visual assessment was the responsibility of 2 crew members. Field crews received 4 weeks of complete protocol training including worm sampling prior to the start of the field season. That training included practice plots and ‘flip-flop’ assessments to ensure standardized calibration among crew members throughout the season. Because the assessment team dug a soil pit in the middle of the plot, measured leaf litter depth and humus depth at the centre of each intensive module while looking for worm evidence, and carefully removed the leaf litter in search of worm evidence, we are confident that the presence of invasive earthworms was accurately recorded (Loss *et al*. 2013). Many species of invasive earthworms are thus included in the field assessment, including but not limited to *Lumbricus terrestris*. We collected about 250 mL of soil for each plot within 10 cm of the soil surface with a soil probe. Soil samples were stored at room temperature for up to 3 months before sending to A&L Great Lakes Laboratories (Fort Wayne, IN). Soil was tested for phosphorus (ppm) and nitrogen (% total). Extractable phosphorus can be broken down into two different forms, phosphorus 1 (‘weak Bray’) and phosphorus 2 (‘strong Bray’). The more commonly used measurement is phosphorus 1, which was developed to correlate with plant-available phosphorus fraction of the soil in slightly acidic soils (Tandon *et al*. 1967). The phosphorus 1 test corresponds with how much phosphorus is available to plants. The phosphorus 2 test gives total soil phosphorus. If there is a big gap between these two numbers, that means much of the phosphorus in the soil is unavailable to plants.

We analysed only plots with information on the presence or absence of *Alliaria petiolata* and/or earthworms to maintain parallel structure to the potted experiment (discussed subsequently). There were a total of 187 plots that met these criteria. Eighty-seven of these plots had zero percent cover of *Podophyllum peltatum*. We then compared the percent cover of *Podophyllum peltatum*, as well as the percent soil nitrogen and two different measures of inorganic phosphorus in parts per million (ppm) across plots with or without these predictor variables (see analysis below).

### Factorial experiment

**Figure 2.**
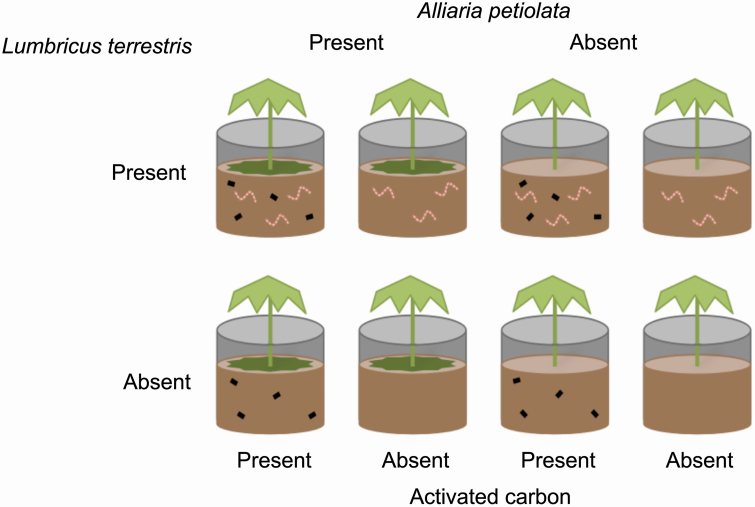
Factorial experimental design assessing the combined effects of invaders on the native plant, *Podophyllum peltatum.* Invasive *Alliaria petiolata* leaves were placed on the soil surface in *Alliaria petiolata* presence treatment, invasive *Lumbricus terrestris* earthworms were placed in pots in the ‘*Lumbricus terrestris* present’ treatment, and activated carbon (black rectangles) was placed in the ‘activated carbon present’ treatment.

The experiment was located at Case Western Reserve University’s Squire Valleevue Farm (‘University Farm’ below) in Hunting Valley, OH, USA (41°29′ N, 81°25′ W) over 2 years (2014–15). The experiment was organized in a 2 × 2 × 2 factorial design, with 6 replicates per experimental treatment, for a total of 48 pots. Each treatment included the presence or absence of the three factors of *Alliaria petiolata*, *Lumbricus terrestris* and activated carbon (Fig. 2). Treatments were randomized within 6 replicate blocks in a common garden. We used 57-L pots (45 cm diameter at top × 42 cm deep). Pots were covered with 30 % shade cloth (Greenhouse Megastore, Danville, IL, USA) to mimic early forest understory conditions in northeastern Ohio. At midday on a sunny day, when ambient PPFD was ~1800, this resulted in an approximate PPFD of 500 under the shade cloth. Plants were kept well-watered throughout the experiment.

On 21 May 2014, 40 L of potting soil (Promix, Quebec, Canada, BX Mycorrhizae general purpose growing medium # 10 381, which uses *Glomus intradices*) were added to each pot, along with a *Podophyllum peltatum* rhizome obtained from Prairie Moon Nursery in Winona, MN and 250 mL of live soil from underneath field *Podophyllum peltatum* to encourage AMF colonization. Initial rhizome size was recorded as length of the rhizome.

Pots with an activated carbon present treatment received 400 g of activated carbon pellets incorporated into the soil evenly distributed through the pots (Lau *et al*. 2008). We used an amount of activated carbon used in previous experiments (Lau *et al*. 2008). To ensure that the *Podophyllum peltatum* would receive adequate nutrients, we added 84 g of nutricote 100-day fertilizer with 18-6-8 N-P-K (#18-6-8NUTRI-100, American Horticultural Supply, Inc.) on 5 June 2014 to all pots, following methods in Hale *et al*. (2011). By adding a small amount of phosphorous, we created conditions where *Podophyllum peltatum* plants should have maintained their AMF mutualism (Hale *et al*. 2011). Fertilizer treatments can influence the effect of activated carbon (Lau *et al*. 2008), so effects of the activated carbon treatment should be interpreted narrowly in this context.

Pots with a *Lumbricus terrestris* present treatment received 16 *Lumbricus terrestris* worms (~100 earthworms per m^2^). This is consistent with natural populations, which can have in excess of 100 individuals per square metre (Eisenhauer *et al*. 2007). We observed earthworms feeding by pulling leaf litter down into the soil, found less leaf cover on top of pots [see **Supporting Information—Fig. S1**], and observed earthworms and their burrows in ‘*Lumbricus terrestris* presence’ treatments at harvest (C.G. Cope, pers. obs), suggesting that our *Lumbricus terrestris* presence treatment was effective in adding earthworms to the experimental plants and initiating leaf litter feeding, as expected. We observed no earthworms or their burrows in *Lumbricus terrestris* absence treatments.

Pots with the *Alliaria petiolata* present treatment were treated weekly with 100 g of fresh *Alliaria petiolata* leaves from second year (bolting) plants of this biennial, spread on the surface of the soil during *Alliaria petiolata*’s growing season, similar to methods in (Brouwer *et al*. 2015). This amount allowed the entire surface of the soil within the pot to be covered to provide substantial leaf litter cover for *Lumbricus terrestris* feeding in treatments containing *Lumbricus terrestris* and make sure that the allelochemicals could leach into all of the soil surface (Brouwer *et al*. 2015). Leaves stayed in place and did not blow away during the experiment, likely because they were wet **[see ****Supporting Information—Fig. S1**, C.G. Cope, pers. obs.].

Once *Podophyllum peltatum* began to senesce in the field in mid-July 2014, the pots containing the *Podophyllum peltatum* rhizomes were stored in an uninsulated barn during the winter to prevent the rhizomes from freezing and dying. The pots were then pulled out of storage in early April 2015. Sixteen worms were again added to *Lumbricus terrestris* present pots, and 100 g of *Alliaria petiolata* was again added weekly to *Alliaria petiolata* present pots in the summer of 2015 until 12 June, when the *Podophyllum peltatum* plants were harvested. Roots were washed and biomass was dried in the drying oven for 24 h (leaves) or 72 h (stems, rhizomes, roots and reproductive) at 60 °C, and weighed to 0.01 g.

### Plant nutrient content and physiology

In order to assess the photosynthetic rate of *Podophyllum peltatum* across experimental treatments, we used an LI-6400XT portable photosynthesis system. Ambient conditions were mostly sunny with an average photosynthetic photon flux density (PPFD) of 1500 µmol m^−2^ s^−1^ and air temperature of ≈21 °C. Conditions within the chamber were maintained at a flow rate of 400 μmol s^−1^, a CO_2_ concentration of 400 μmol mol^−1^, a leaf temperature of 21.4 °C, and between 40 and 50 % relative humidity. PPFD was set to 1200 μmol m^−2^ s^−1^ for all individuals measured. Based on previous data (not presented), this light level represented saturation for plants without inducing photoinhibition. Once plants stabilized in the chamber we logged 3 data points per plant on 23 May 2015 between 11:00 AM and 1:00 PM before rates began to lose stability.

In order to assess leaf nutrient content, we followed the methods described in Khasanova *et al*. (2013) for CN analysis. Plant biomass was washed with DI water and oven-dried at 65 °C. Subsamples of dried plant tissue were ground with a ball mill, then 2 ± 0.1 μg samples were weighed for total N and C concentration by micro Dumas combustion on a CN analyzer (Costech Analytical, Valencia, CA).

To determine AMF colonization, we stained roots collected from the *Podophyllum peltatum* plants at the end of the experiment. This procedure followed root staining protocol put forth by McGonigle and colleagues where roots were cleared for 20 min at 121 °C in a 10 % KOH solution, and stained in Chlorazol Black E (McGonigle *et al*. 1990). Following staining we examined the stained roots under a microscope [digital camera (Model #C-3040; Olympus America Inc., Melville, NY, USA) attached to a microscope (Model #BH-2; Olympus Corporation, Lake Success, NY, USA)] at ×40 and ×100 magnification. We found few traces of any AMF colonization on any of the 47 roots examined (one plant died before the end of the experiment). Seven out of 47 pots had some signs of colonization and there was no apparent pattern with respect to treatment. We also performed DNA extractions on both ground and unground roots, and used polymerase chain reaction (PCR) using the AMG1F and AM1 primers to amplify any AMF DNA found within the *Podophyllum peltatum* roots. We then used gel electrophoresis to determine whether there was AMF colonization of our samples. There were no visible bands on any of the 41 roots examined (a few roots did not yield strong DNA extractions), indicating that the *Podophyllum peltatum* roots had no detectable AMF DNA present.

### Statistical analysis

#### Plant community assessment program—observational data.

To ensure the predictors in the model below were statistically independent, we tested whether soil nutrients (% soil nitrogen, phosphorus 1, phosphorus 2) predicted *Alliaria petiolata* percent cover in the observational data set. We used three separate linear models with log transformed *Alliaria petiolata* percent cover—with a small constant (0.001) added before transformation to retain zero values in the data—as a function of log transformed % soil nitrogen, phosphorus 1 or phosphorus 2. This is important because *Alliaria petiolata* may directly influence soil nutrient availability (Rodgers *et al*. 2008). Further, we also explored whether *Alliaria petiolata* presence, invasive earthworm presence or their interaction predicted soil nutrient availability for % soil nitrogen, phosphorus 1 and phosphorus 2 in separate linear models. Soil nitrogen and phosphorus were log transformed to normalize the data. Because we used observational data, the ‘design’ of this analysis was unbalanced. Therefore, we used ‘type II’ sums of squares (Herr 1986), using the drop1 function in R.

In addition, we conducted a contingency table analysis and found that the presence of one invader did not influence the presence of the other (χ ^2^ = 1.53, df = 1, *P* = 0.22). Thus, we treated the presence of *Alliaria petiolata* and earthworms as independent variables in the following analysis. We used the presence/absence of *Alliaria petiolata* (rather than percent cover) for parallel structure with the experimental design, described below. Next we modelled percent cover of *Pododphyllum peltatum* as a response variable in a linear model with a Guassian error distribution and log transformed percent cover, with a small constant added (0.001) to prevent zero values of percent cover from being dropped from the model. We included predictors: *Alliaria petiolata* presence, earthworm presence, soil nitrogen and all of their interactions. Because our analyses indicated that soil phosphorus was not independent of earthworms, we did this analysis only for soil nitrogen **[see **Supporting Information—**Table S1****]**. We used stepAIC to select a minimal model (Venables and Ripley 2002) and we used the drop1 function in R to test for significance.

#### Factorial experiment.

We fit a linear model with *Podophyllum peltatum* final dry biomass as a response variable and *Alliaria petiolata, Lumbricus terrestris* and activated carbon treatments, and their interactions as predictors. We used log-transformed final biomass and a Gaussian error distribution. We then conducted planned contrasts within both the *Lumbricus terrestris* and *Alliaria petiolata* treatments. We explored a model with initial rhizome size (length) as a covariate. Initial size was not predictive of final mass after 2 years in the pot experiment (*F*_1,38_ = 0.11, *P* = 0.74), and was, therefore, not included in the final models. Block was not significant in initial models (*F*_1,38_ = 0.71, *P* = 0.48), and was not included in final models.

For our physiological measures, C_i_ and A_max_, we modelled untransformed variables as a function of *Alliaria petiolata, Lumbricus terrestris* and activated carbon treatments, and their interactions as predictors. Then we used model selection by AIC, using the function stepAIC in the MASS package (Venables and Ripley 2002), to select a minimal model.

All datasets were examined for potential outliers as well as tested for normality. Q-Q norm and residual vs. fitted plots were examined for each model to check model assumptions (Bolker *et al*. 2008). All model assumptions were met. All analyses were conducted in the R statistics program (version 3.4.3, R Core Development Team 2014).

## Results

### Plant community assessment program observational data

There was no evidence in the observational data for a direct interaction between invasive earthworm presence and *Alliaria petiolata* on cover of the native plant, *Podophyllum peltatum* (Table 1). The presence of *Alliaria petiolata* and high concentrations of soil nitrogen interacted in a model of *Podophyllum peltatum* cover, with the least *Podophyllum peltatum* cover in *Alliaria petiolata* sites with high soil nitrogen (Table 1, **see**  **Supporting Information—Fig. S2**). Soil phosphorus predicted *Alliaria petiolata* abundance across the field plots **[see**** Supporting Information—Table S1****]**, indicating that *Alliaria petiolata* and soil phosphorus could not be used as independent predictors of *Podophyllum peltatum* cover. Thus, we included only soil nitrogen as a predictor in models of *Podophyllum peltatum* cover (Table 1).

**Table 1.** Observational field data, modelling the interaction of invasive *Alliaria petiolata*, invasive earthworm presence and soil nitrogen on the percent cover of native Podophyllum peltatum*.

	DF	Deviance	AIC	*F*-value	*P*-value
*Alliaria petiolata* presence/absence	1	1726	962.25	2.46	0.12
earthworm presence/absence	1	1713	960.88	1.14	0.29
Total Soil Nitrogen (%)	1	1745	964.41	4.56	0.03
*Alliaria petiolata* × earthworm	1	1715	961.17	1.41	0.24
*Alliaria petiolata* × Nitrogen	1	1742	964.10	4.27	0.04*
earthworm × Nitrogen	1	1712	960.76	1.02	0.31
*Alliaria petiolata* × earthworm × Nitrogen	1	1724	962.03	2.25	0.14

*An alternative model excluding zero values for percent cover of *Podophyllum peltatum* found only a marginally significant interaction here (*F*_1,373_ = 2.98, *P* = 0.087).

We also asked whether the presence of these invasive species predicted soil nitrogen or phosphorus. The presence of earthworms predicted lower total soil nitrogen **[see**  Supporting Information—**Table S2**, **Fig. S3a****]**. We also found that *Alliaria petiolata* and earthworm presence interacted in a model of phosphorus 1 in the soil, with the lowest phosphorus available when *Alliaria petiolata* was absent and earthworms were present **[see**  Supporting Information—**Fig. S3b****]**. Phosphorus 2 availability was not correlated with these invaders **[see **Supporting Information—**Table S2****]**.

### Factorial experiment

**Figure 3.**
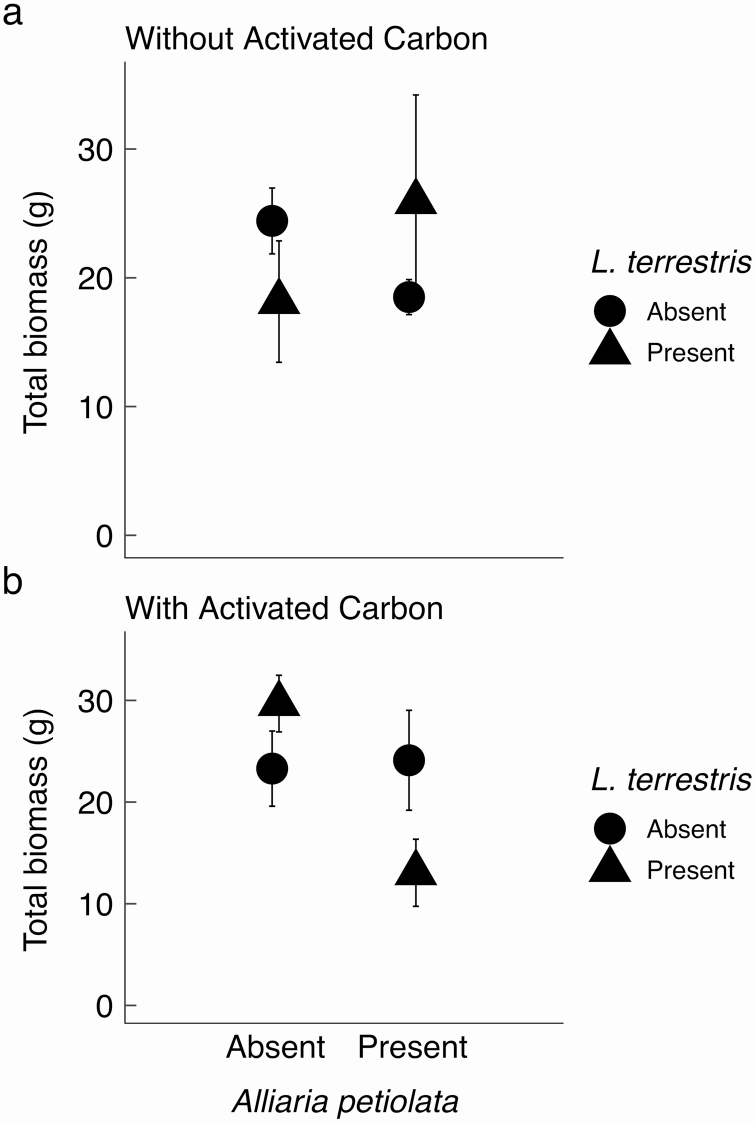
The effect of invasive *Lumbricus terrestris* earthworms and invasive *Alliaria petiolata* leaf litter on native *Podophyllum peltatum* total biomass in a factorial experiment. (a) In the absence of activated carbon, there were no differences in *Podophyllum peltatum* biomass across treatments. (b) In the presence of activated carbon, *Podophyllum peltatum* biomass was significantly reduced by the presence of both earthworms and *Alliaria petiolata* [see Supporting Information— Tables S3 and S4]. Means ± 1 SE.

Overall, we found some evidence for a potential interaction between the invasive earthworm, *Lumbricus terrestris* and *Alliaria petiolata* leaf litter presence, though there was no direct evidence that this interaction was driven by allelopathy. We observed no colonization of *Podophyllum peltatum* roots by AMF in any experimental treatment, either via staining or qPCR. We found that the total biomass of *Podophyllum peltatum* was significantly reduced when *Lumbricus terrestris* earthworms, *Alliaria petiolata* and activated carbon were present (Table 2, Fig. 3), contrary to the prediction of an allelopathy effect in the absence of activated carbon. Contrasts showed a significant negative effect of *Alliaria petiolata* on *Podophyllum peltatum* biomass when both *Lumbricus terrestris* and activated carbon were present (*P* = 0.007, **see**  Supporting Information—**Table S3**, Fig. 3). Further we found a marginally significant effect of *Lumbricus terrestris* on *Podophyllum peltatum* biomass in the presence of *Alliaria petiolata* (*P* = 0.07, **see ****Supporting Information—Table S4**, Fig. 3).

**Table 2.** Total plant biomass of native *Podophyllum peltatum* in response to experimental treatments (see contrasts in Supporting Information—Tables S3 and S4).

	DF	Deviance	AIC	*F*-value	*P*-value
*Alliaria petiolata*	1	4090.8	359.30	1.03	0.32
Activated carbon (AC)	1	3989.6	358.12	0.04	0.85
*Lumbricus terrestris*	1	4103.5	359.44	1.15	0.29
*Alliaria petiolata* × AC	1	4053.9	358.87	0.67	0.42
*Alliaria petiolata* × *Lumbricus terrestris*	1	4253.1	361.13	2.62	0.11
AC × *Lumbricus terrestris*	1	4226.2	360.83	2.35	0.13
*Alliaria petiolata* × AC × *Lumbricus terrestris*	1	4695.3	365.78	6.94	0.01

**Figure 4.**
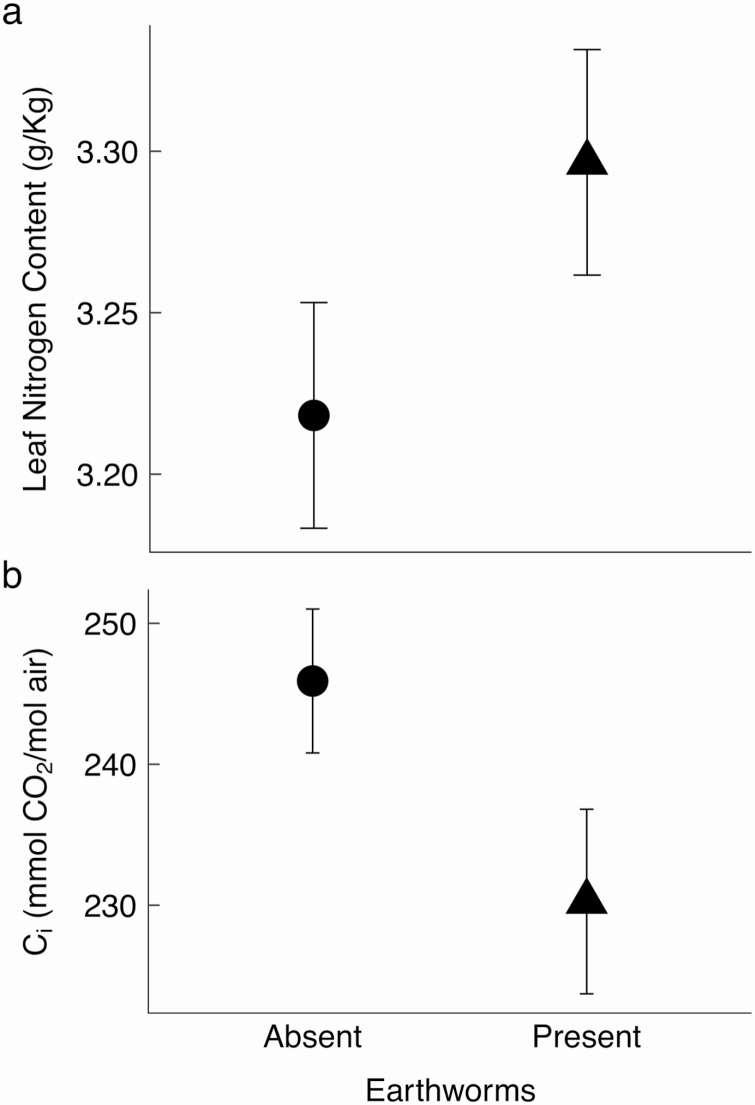
(a) Leaf nitrogen content of *Podophyllum peltatum* was greater in the presence of invasive *Lumbricus terrestris* earthworms (*F*_1,1_ = 4.16, *P* = 0.05) in a pot experiment. (b) Internal leaf CO_2_ concentration (C_i_) of *Podophyllum peltatum* was reduced when earthworms were present (Table 3). Means ± 1SE

*Podophyllum peltatum* leaf nutrient content and internal CO_2_ concentration were both influenced by experimental treatments. We found leaf nitrogen content was significantly increased in the presence of *Lumbricus terrestris* (Fig. 4a). There was also a significant decrease of internal CO_2_ concentration (C_i_) within the leaves of *Podophyllum peltatum* when in the presence of *Lumbricus terrestris* (Table 3, Fig. 4b). There was a marginally significant reduction in photosynthetic rate when both *Alliaria petiolata* and activated carbon were present (Table 4), and *Alliaria petiolata* alone resulted in only a 1 % reduction in photosynthetic rate. Further, there was no effect of *Lumbricus terrestris* on photosynthetic rate (Table 4).

**Table 3.** C_i_, the intercellular CO_2_ concentration in leaves, as a function of experimental treatments on native plant *Podophyllum peltatum.* Model was chosen by AIC.

	DF	Deviance	AIC	*F*-value	*P*-value
*Alliaria petiolata*	1	26 255	430.31	0.13	0.72
Activated carbon (AC)	1	26 339	430.45	0.25	0.62
*Lumbricus terrestris*	1	30 764	437.44	6.51	0.01
*Alliaria petiolata* × AC	1	26 372	430.51	0.30	0.59
*Alliaria petiolata* × *Lumbricus terrestris*	1	27 984	433.18	2.58	0.12
AC x *Lumbricus terrestris*	1	27 782	432.85	2.29	0.14
*Alliaria petiolata* × AC × *Lumbricus terrestris*	1	27 562	432.49	1.98	0.17

**Table 4.** *Podophyllum peltatum* photosynthesis rate (A_max_) as a function of *Alliaria petiolata* and activated carbon experimental treatments. Minimal model chosen by AIC. Earthworm presence/absence was dropped from the model as this treatment had no effect on photosynthetic rate.

	DF	Deviance	AIC	*F*-value	*P*-value
*Alliaria petiolata*	1	0.75	184.97	1.23	0.27
Activated carbon (AC)	1	0.71	184.85	1.12	0.30
*Alliaria petiolata* × AC	1	1.07	187.05	3.23	0.08

## Discussion

Because relatively few studies have examined effects of multiple invaders on native plants, whether such relationships are largely additive, or sub- or super-additive is still not fully understood (Braga *et al*. 2018). We find evidence consistent with the possibility of super-additive effects (Braga *et al*. 2018), but only in some experimental contexts. The interaction only in the presence of activated carbon, combined with the lack of AMF colonization in any experimental treatment, suggests that the experimental interaction between *Alliaria petiolata* and *Lumbricus terrestris* was not due to an indirect effect of allelochemicals on AMF (Fig. 1, arrow 2), contrary to indirect effects of *Alliaria petiolata* allelochemical via AMF in other systems (Hale *et al*. 2016). Our experimental plants may have lacked AMF colonization because they were large healthy rhizomes at the beginning of the experiment, though this is speculative. Although *Podophyllum peltatum* has not been reported in the field to have facultative AMF association (Soudzilovskaia *et al*. 2020), the ability to ‘cut off’ mycorrhizal partners is well-described and common in many plant species (Johnson *et al*. 1997). In the field, *Podophyllum peltatum* cover was lowest at sites that had both greater soil nitrogen and *Alliaria petiolata* present, and the presence of earthworms reduces soil nitrogen content in the field and increases leaf nitrogen content in the experiment. If earthworms speed up nutrient cycling (Costello and Lamberti 2009), there might be less nitrogen in the soil and more within plant tissues. This increase in nutrients within the plant might reduce some of the harmful effects *Alliaria petiolata* has on *Podophyllum peltatum*, though our survey data suggest that such effects are likely weak. In addition, like others before us, we found no evidence for a correlation between earthworms and garlic mustard in the field (Zelles 2012), [however, see Nuzzo *et al*. (2009)], inconsistent with an ‘invasion meltdown’ hypothesis of these two invaders facilitating one another (Simberloff and Von Holle 1999).

*Alliaria petiolata* has the potential for both direct phytotoxic effects (Cipollini *et al*. 2012; Cipollini and Bohrer 2016) and indirect effects via AMF mutualists (Cipollini and Bohrer 2016; Hale *et al*. 2016). However, there is also mixed evidence that *Alliaria petiolata* has any direct effects on abundance in the field (Davis *et al*. 2015). Contrary to some previous work (Davis *et al*. 2015), we find a strong negative effect of *Alliaria petiolata* presence on native *Podophyllum peltatum* abundance in the field, when soil nitrogen levels are low, consistent with either competition or allelopathy (Cipollini and Cipollini 2016). Our results are consistent with studies of the demography of native plant *Trillium erectum*, whose population growth rate is suppressed by *Alliaria petiolata* in the field (Bialic-Murphy *et al*. 2020). Generally, more work is needed to disentangle direct and indirect effects of invaders like *Alliaria petiolata* on native plants (Cipollini and Cipollini 2016). Our work contributes to this line of research by suggesting that soil nutrient-mediated effects may be as or more important than allelopathy, at least for this native plant (Fig. 1), and that these effects can occur even in the absence of AMF mutualists and may depend on nutrient availability in the soil.

Both our experiment and field observational data show that changes in nutrient acquisition or soil conditions can influence the strength or presence of the interaction between these invaders. While adding activated carbon to our experiment was intended to neutralize allelochemicals from *Alliaria petiolata*, effects of activated carbon on soil nutrient availability and plant performance are complex (Lau *et al*. 2008), and activated carbon may have acted as an additional stressor to the native plant—e.g. through reduced ammonium availability (Lau *et al*. 2008) or changes to the soil microbial community (Weisshuhn and Prati 2009). Too much nitrogen can lead to excess leaf production at the cost of root production, leaving plants vulnerable to drought (Templer 2013). Stress caused by activated carbon in this experiment may have caused *Podophyllum peltatum* to be more vulnerable to the harmful effects of the worms and *Alliaria petiolata*, which resulted in reduced *Podophyllum peltatum* biomass; however, the mechanisms that govern this effect are unknown. In the field study, both earthworms and *Alliaria petiolata* were a significant function of soil nutrients. This suggests that significant decreases in *Podophyllum peltatum* cover or biomass due to earthworm and *Alliaria petiolata* invasion may be context-dependent and influenced by nutrient availability within the soil.

Plant responses to multiple invaders are likely to be mediated by plant physiology, as others have seen for responses to single invaders, like *Alliaria petiolata* (Brouwer *et al*. 2015; Hale *et al*. 2016). In prior studies on native herbaceous perennial *Maianthemum racemosum*, *Alliaria petiolata* has been shown to reduce carbon storage by suppressing fungal mutualists (Brouwer *et al*. 2015), reducing the native plant’s photosynthetic rate (Hale *et al*. 2016). We found no AMF colonization in our experimental plants, and no direct effects of *Alliaria petiolata* on native plant physiology. Here, we found that the internal CO_2_ concentration in the leaves was reduced in the presence of the invasive earthworm *Lumbricus terrestris*. This suggests that *Lumbricus terrestris* alters plant physiology by changing nutrient availability. If these invasive earthworms increase nitrogen availability to the plant, this could influence *Podophyllum peltatum* production or activation of RuBisCO, the enzyme used to fix CO_2_ in the leaf (Spreitzer and Salvucci 2002). The higher leaf nitrogen content in the presence of *Lumbricus terrestris* is consistent with this explanation and the observation that *Lumbricus terrestris* enhances soil nutrient cycling (Costello and Lamberti 2009). This could increase the rate of CO_2_ usage, decreasing internal CO_2_ concentrations (C_i_). Therefore, if stomatal conductance stays the same, lower C_i_ could indicate greater carbon drawdown within the leaf. This makes sense if earthworms like *Lumbricus terrestris* make nitrogen more available to the plants, increasing leaf RuBisCO content and thus drawdown capacity. However, this faster C_i_ drawdown does not lead to greater net photosynthetic rates. A possible reason for this could be that photosynthetic rates are limited not just by RuBisCO availability, but also by the rate sugars move through the plant. If there is a backup of sugars near the photosynthetic machinery, there could be a reduction in photosynthetic rates (Brodribb *et al*. 2007). In sum, *Lumbricus terrestris* directly impacts *Podophyllum peltatum* physiology (lower CO_2_ concentration in the leaf), but does not lead to improvements in plant performance (e.g. greater maximum photosynthetic rate or plant biomass).

Greater nutrient cycling by invasive earthworms like *Lumbricus terrestris* (Costello and Lamberti 2009) combined with *Alliaria petiolata* leaves (Rodgers *et al*. 2008), harms the native plant *Podophyllum peltatum* in the absence of AMF, at least under some soil conditions. Additional experiments with non-*Alliaria petiolata* leaf litter controls might help distinguish between allelopathy and nutrient effects as mechanisms (Fig. 1) (Hale *et al*. 2011). While our observational data in the field suggests that these two invaders might not be interacting currently to suppress *Podophyllum peltatum* cover, our experiment suggests that such interactions are likely, given sufficient stress on native plants. As environmental stressors, such as nutrient deposition (Fowler *et al*. 2013) and climate change (IPCC 2014), increase, the potential for such negative super-additive effects between invaders may also increase (Mooney and Hobbs 2000). We suggest that field experiments that manipulate multiple stressors simultaneously, including nutrient deposition and multiple invaders, are needed to fully understand multi-invader interactions and their context-dependence. Our data suggests that even plants with positive physiological responses, like increased CO_2_ drawdown in the leaf with increased nitrogen availability, are not able to compensate for the harm caused by multiple invaders.

## Supporting Information

The following additional information is available in the online version of this article—

**Table S1.** Three separate linear models testing the effects of soil nutrients on the percent cover of *Alliaria petiolata* in observational field data.

**Table S2.** The interaction between invasive *Alliaria petiolata* and invasive earthworms on soil nitrogen, phosphorus 1 and phosphorus 2 in observational field data.

**Table S3.** Contrasts for *Alliaria petiolata* effect, contrasting *Alliaria petiolata* presence to *Alliaria petiolata* absence treatments, either in the presence or absence of *Lumbricus terrestris* earthworms and activated carbon (AC) in the soil.

**Table S4.** Contrasts for earthworm effect, contrasting earthworm presence to earthworm absence treatments, either in the presence or absence of *Alliaria petiolata* and activated carbon in the soil.

**Figure S1**. Experimental *Podophyllum peltatum* plants, in pots with *Alliaria petiolata* leaves added.

**Figure S2.** The interaction between soil nitrogen and *Alliaria petiolata* presence correlated with *Podophyllum peltatum* cover in the 187 observational field plots (Table 1).

**Figure S3.** Supplemental field data.

plaa073_suppl_Supplementary_Materials

## Data Availability

Data are available at the Open Science Framework. Doi: 10.17605/OSF.IO/FTBYG

## Code availability

DOI 10.17605/OSF.IO/FTBYG

## Notes

**Populations & Communities Chief Editor:** Jean Burns
