# Supplementary material for: Potential interactive effects between invasive Lumbricus terrestris earthworms and the invasive plant Alliaria petiolata on a native plant Podophyllum peltatum in northeastern Ohio, USA
Source: AoB Plants. 2020 Dec 29;13(1):plaa073. doi: 10.1093/aobpla/plaa073 (PMC7877696; doi:10.1093/aobpla/plaa073)
Supplement: plaa073_suppl_Supplementary_Materials [file plaa073_suppl_supplementary_materials.pdf]

Appendix A

Table S1 Three separate linear models testing the effects of soil nutrients on the percent cover of *Alliaria petiolata* in observational field data

	DF	Deviance	Residual Deviance	F-value	p-value
Total Soil Nitrogen	1	7.99	1791.60	0.82	0.37
Phosphorus 1	1	117.40	1682.20	12.77	0.0004
Phosphorus 2	1	247.47	1552.10	29.18	<0.001

Table S2 The interaction between invasive *Alliaria petiolata* and invasive earthworms on soil nitrogen, phosphorus 1, and phosphorus 2 in observational field data

**Total Soil Nitrogen**	**DF**	**Deviance**	**AIC**	**F-value**	**p-value**
Alliaria petiolata presence/absence	1	15.67	75.00	0.73	0.39
earthworm presence/absence	1	15.94	78.27	3.98	0.05
Alliaria petiolata x earthworm	1	15.78	76.32	2.04	0.16
**Phosphorus 1**					
Alliaria petiolata presence/absence	1	86.63	394.80	0.49	0.48
earthworm presence/absence	1	88.50	398.78	4.44	0.04
Alliaria petiolata x earthworm	1	87.79	397.28	2.94	0.09
**Phosphorus 2**					
Alliaria petiolata presence/absence	1	76.32	371.11	1.65	0.20
earthworm presence/absence	1	76.88	372.45	2.98	0.09
Alliaria petiolata x earthworm	1	75.49	371.52	2.05	0.15

Table S3 Contrasts for *Alliaria petiolata* effect, contrasting *Alliaria petiolata* presence to *Alliaria petiolata* absence treatments, either in the presence or absence of *Lumbricus terrestris* earthworms and Activated Carbon (AC) in the soil

**Within treatment categories:**	**t-value**	**p-value**
**Activated carbon absent**		
Lumbricus terrestris absent	1.00	0.32
Lumbricus terrestris present	-1.27	0.21
**Activated carbon present**		
Lumbricus terrestris absent	-0.14	0.89
Lumbricus terrestris present	2.85	0.007

Table S4 Contrasts for earthworm effect, contrasting earthworm presence to earthworm absence treatments, either in the presence or absence of *Alliaria petiolata* and activated carbon in the soil

	**t-value**	**p-value**
**Activated carbon absent**		
Alliaria petiolata absent	1.07	0.29
Alliaria petiolata present	-1.21	0.23
**Activated carbon present**		
Alliaria petiolata absent	-1.10	0.28
Alliaria petiolata present	1.90	0.07

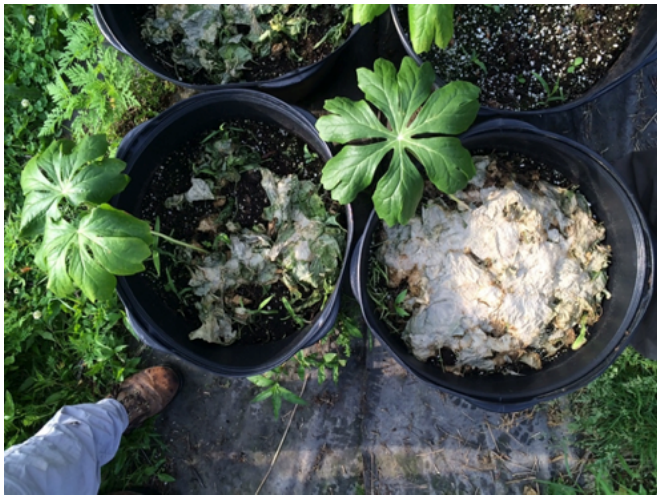

Figure S1. Experimental *Podophyllum peltatum* plants, in pots with *Alliaria petiolata* leaves added. The pot on the left had *Lumbricus terrestris* earthworms added. The pot on the right did not have earthworms added. We observed *Lumbricus terrestris* earthworms pulling leaf litter down into the soil and observed burrows and earthworms at the end of the experiment only in the *Lumbricus terrestris* presence treatment.

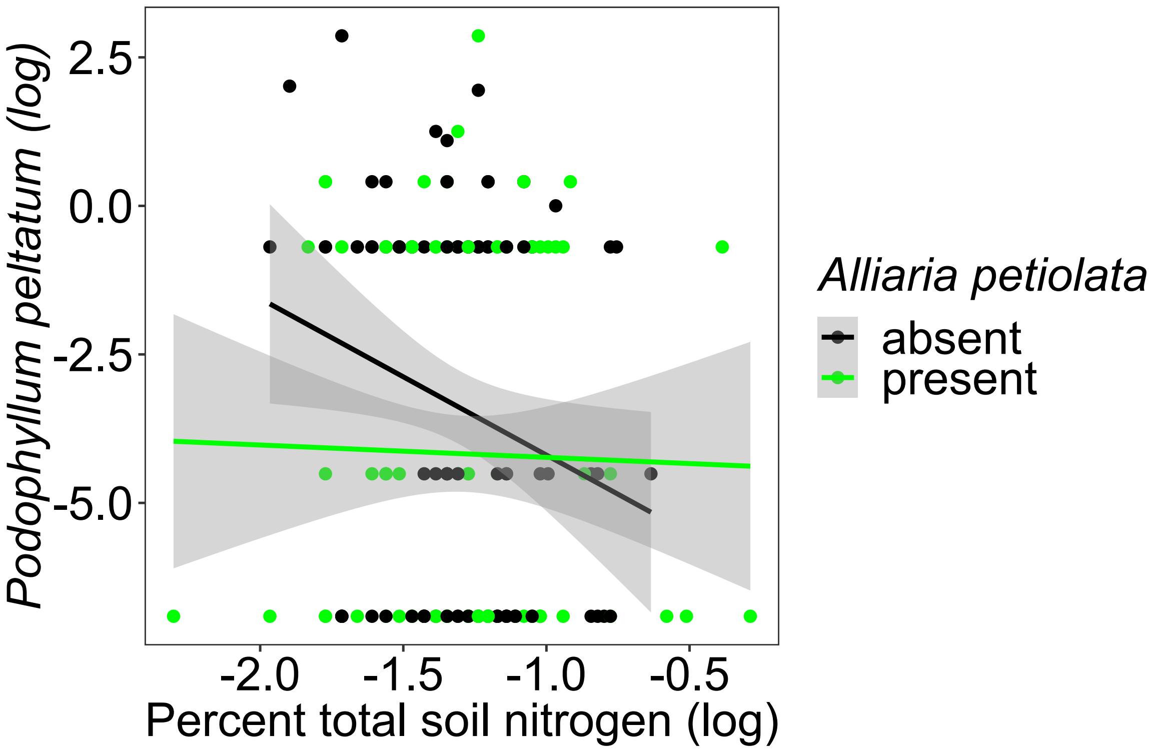

Figure S2 The interaction between soil nitrogen and *Alliaria petiolata* presence correlated with *Podophyllum peltatum* cover in the 187 observational field plots (Table 1). *Podophyllum peltatum* cover was lower in field plots when nitrogen in the soil is higher and when *Alliaria petiolata* is present (green circles) versus when it was absent (black circles) and was highest for plots with low soil nitrogen and without *Alliaria petiolata*. Of these plots, 87 contained estimates of *Podophyllum peltatum* cover of zero.

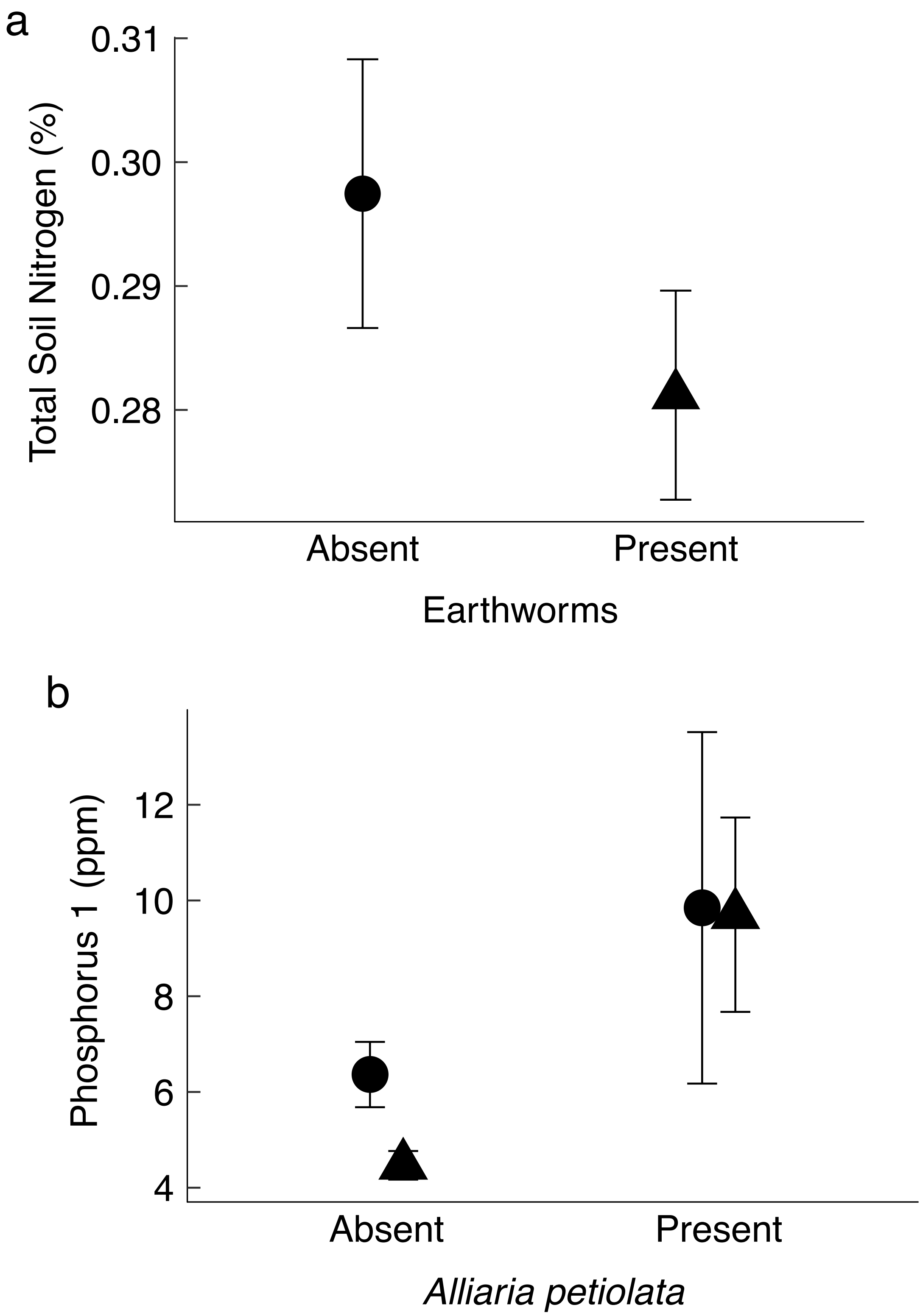

Figure S3 (a) Percent soil nitrogen was significantly lower in plots that had earthworms present when compared to plots without earthworms (Table S2). (b) The interaction between earthworms and *Alliaria petiolata* on the amount

of phosphorus within the soil in observational data in the field (Table S2). Circles = earthworms absent and triangles = earthworms present. Means ± 1 SE.
